# Obesity Treatment Pathways and Implementation in Health Systems

**DOI:** 10.1002/osp4.70142

**Published:** 2026-04-20

**Authors:** Ariana M. Chao, Stephanie Walsh, Pamela R. Rama, Jason M. Samuels

**Affiliations:** ^1^ Johns Hopkins University School of Nursing Baltimore Maryland USA; ^2^ ProCare Telehealth Intero Health Atlanta Georgia USA; ^3^ Baptist Health Cardiometabolic Clinic Jacksonville Beach Florida USA; ^4^ Section of Surgical Sciences Vanderbilt University Medical Center Nashville Tennessee USA

**Keywords:** adult, care pathways, delivery of health care, evidence‐based practice, obesity management

## Abstract

**Background:**

Although clinical practice guidelines are valuable for managing obesity and should be the foundation for health system protocols for screening, diagnosis, and treatment, these guidelines need to be translated and implemented within clinical care pathways that account for organizations' processes, structures, and cultures. Variation in resources across systems is another challenge.

**Methods:**

A narrative review was undertaken to assess how obesity treatment pathways can support health systems and HCPs in informed decision‐making for obesity management, and to offer practical considerations for developing and implementing health care system treatment pathways.

**Findings:**

Little publicly available information exists on developing and implementing obesity treatment pathways in clinical practice within health systems. This review discusses the key elements of obesity care, including screening, diagnosis, treatment, and monitoring, and outlines the roles and responsibilities of health care professionals within each step of the pathway.

**Conclusion:**

This review offers resources and considerations for developing and implementing obesity care pathways in primary care, specialty care, and various other settings to assist health systems and health care professionals in making informed, evidence‐based decisions in obesity management.

## Graphical abstract

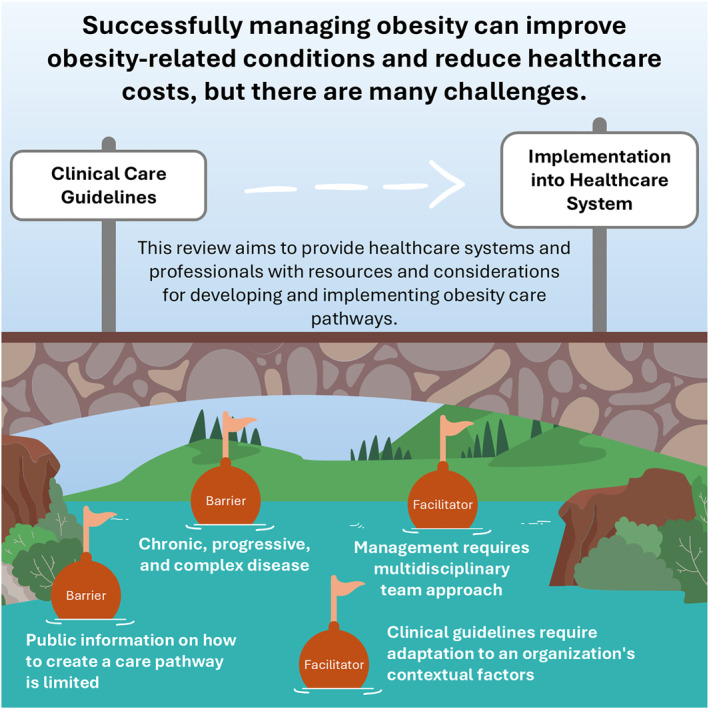

## 1 Introduction

Clinical practice guidelines are valuable for health care professionals (HCPs) in managing obesity and can help serve as the foundation for health system protocols for obesity screening, diagnosis, and treatment. Spurred, in part, by growing investigations and indications of obesity therapies for improving and/or treating obesity‐related conditions, an increasing number of clinical practice specialties have developed guidelines for obesity management in adults and children/adolescents [1, 2, 3, 4, 5, 6, 7, 8]. This highlights the importance of obesity management not only for improving adiposity but also for addressing obesity‐related conditions, and recognizing that obesity is not solely the domain of primary care professionals or any one medical specialty.

Treatment care pathways can help guide the implementation of evidence‐based health care, aiming to “translate clinical practice guideline recommendations into clinical processes of care within the unique culture and environment of a health care institution,” increasing efficiency, decreasing costs, and improving quality [9, 10]. Clinical care pathways have been developed for other disease states or conditions, including oncology and mental health, improving patient care and outcomes [11, 12, 13, 14]. For obesity, procedure‐specific care pathways exist for bariatric surgery in adults and adolescents [15, 16]. Although frameworks have been proposed for treatment pathways and standards of care in obesity [17, 18], they were developed primarily before the widespread use, uptake, and expanding indications for glucagon‐like peptide‐1 receptor agonists (GLP‐1RAs) [19]. Information on developing and implementing obesity care pathways in clinical practice within health systems is limited, and the resources available across health systems vary considerably. Furthermore, holistic pathways are needed for the management of obesity, extending from primary care to secondary care services [20].

Clinicians and health care systems face challenges such as managing drug titrations, tackling potential obstacles to help patients mitigate risks of side effects, addressing insurance requirements and prior authorizations, and the need for long‐term treatment to optimize health outcomes; no care pathways exist to guide these aspects of care. Our solution to these challenges is to suggest practical considerations for developing and implementing health care system treatment pathways that can be flexibly used in primary and specialty care and various settings (e.g., rural, urban, and academic medical centers). This review describes how obesity treatment pathways can support health systems and HCPs in informed decision‐making for obesity management.

## 2 Benefits and Key Considerations of Care Pathways

Benefits of care pathways include standardization, shared documentation, evaluation tools, and enhanced communication [11, 21, 22]. Protocol‐based, standardized clinical and administrative roadmaps define care management and workflows (e.g., ordering and reviewing laboratory results to monitor obesity‐related conditions to eliminate redundancies, up and down titrating medications), reduce confusion, and improve structure, efficiency, quality, and outcomes [9, 10]. Although pathways specify the ordering of key elements in the care continuum, it is also critical to understand the roles and responsibilities of each HCP at each step in the pathway from screening to diagnosis, treatment, and follow‐up.

## 3 Key Elements of a Clinical Treatment Pathway for Obesity

The key aspects of a clinical treatment pathway for obesity include screening and assessing the patient, diagnosing, working with other specialists to manage obesity and/or obesity‐related conditions, collaborating with multidisciplinary stakeholders to address diet, physical activity, and behavior modifications, prescribing and managing pharmacotherapy, engaging with MBS teams pre‐, intra‐, and postoperatively, managing other treatments and conditions, and engaging payers to ensure coverage of evidence‐based therapies. Most North American clinical practice guidelines for children, adolescents, and adults provide guidance for screening, addressing obesity‐related conditions, lifestyle modifications, pharmacotherapy, and bariatric surgery, but many lack recommendations for making a formal diagnosis or documenting in patients' health records (Table 1) [1, 2, 3, 4, 5, 6, 8, 23, 24, 25, 26].

**TABLE 1:** Summary of obesity clinical practice guidelines.

Obesity clinical practice guidelines (US and Canada)								
	American Heart Association/American College of Cardiology/The Obesity Society (AHA/ACC/TOS) [1]	Endocrine Society [2]	American Association of Clinical Endocrinologists/American College of Endocrinology (AACE/ACE) [3]	Department of Veterans Affairs/Department of Defense [23]	Obesity Canada/Canadian Association of Bariatric Physicians and Surgeons [6]	American Gastroenterological Association (AGA) [4, 24]	American Academy of Pediatrics [8]	American Diabetes Association (ADA) [5]
Year published	2013	2015	2016	2020	2020	2017, 2022^a^	2023	2025
Population addressed	General population of adults	General population of adults	General population of adults, adolescents, and children	General population of adults	General population of adults	General population of adults General population of adults, adolescents, and children	General population of children and adolescents aged 2–18 years	General population of adults
Screening for obesity	BMI (annually or more often) Waist circumference (annually or more often)	No specific assessments/timing mentioned	BMI (annually) Waist circumference (patients with BMI < 35 kg/m^2^)	BMI (annually) Waist circumference (consider for patients with BMI 25–29.9 kg/m^2^)	BMI (regularly) Waist circumference (regularly assess patients with a BMI 25–35 kg/m^2^)	BMI (no timing mentioned) Waist circumference or waist‐to‐hip ratio (no timing mentioned)	BMI (annually) BMI percentile using age‐ and sex‐specific CDC growth charts (all children aged 2–18 years, annually)	BMI (annually) Additional measurements of body fat distribution, such as waist circumference, waist‐to‐hip ratio, and/or waist‐to‐height ratio if BMI is indeterminate
Formal obesity diagnosis	No	No	Yes	No	No	No	Yes	Yes
Address associated metabolic conditions	Yes	No	Yes	Yes	Yes	Yes	Yes	Yes
Recommendations for lifestyle interventions	Reduced‐calorie diet Increased physical activity Comprehensive behavioral modification program with counseling	Not included	Reduced‐calorie diet Increased physical activity Comprehensive behavioral modification program with counseling	Reduced‐calorie diet Increased physical activity Various interventions, including self‐monitoring, behavioral modification, and cognitive strategies	Personalized dietary recommendations Increased physical activity Various interventions, including behavioral modification and cognitive strategies	Reduced‐calorie diet Increased physical activity Various interventions, including self‐monitoring, behavioral modification, cognitive strategies, and counseling	Intensive health behavior and lifestyle modification	Reduced‐calorie diet Increased physical activity Intensive behavioral modification program with counseling
Recommendations for pharmacotherapy	Little mention, as few drugs were available at the time of publication	Adjunct to lifestyle modifications First‐generation AOMs	Adjunct to lifestyle modifications First‐generation AOMs	Adjunct to lifestyle modifications First‐generation AOMs	Adjunct to lifestyle modifications	Adjunct to lifestyle and recommended to add in patients with complications versus lifestyle modifications alone First‐ and second‐generation AOMs	Adjunct to lifestyle Second‐generation AOMs	Pharmacotherapy recommended with lifestyle modification First‐ and second‐generation AOMs GLP‐1RA preferred therapy for patients with overweight/obesity and type 2 diabetes
Recommendations for metabolic and bariatric surgery	Yes	No	Yes	Yes	Yes	Yes	Yes	Yes

Abbreviations: AOMs, anti‐obesity medications; BMI, body mass index; CDC, Centers for Disease Control and Prevention; GLP‐1RA, glucagon‐like peptide‐1 receptor agonist.

^a^ Pharmacological interventions specifically.

### 3.1 Screening and Assessing Patients for Obesity and Making a Diagnosis

**FIGURE 1:**
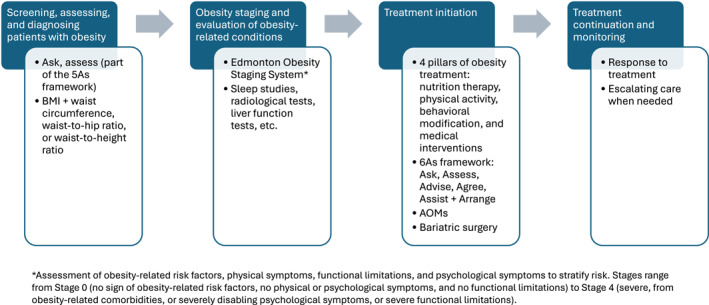
Key elements of a clinical treatment pathway for obesity. AOM, anti‐obesity medication; BMI, body mass index.

Opportunities to assess patients for obesity include annual physical exams, appointments specifically targeted for obesity treatment, and evaluation or treatment for an obesity‐related condition (Figure 1). In a clinical care pathway, various HCPs play a role in assessing obesity, including primary care providers and specialists such as cardiologists, gastroenterologists, hepatologists, endocrinologists, and sleep specialists; any of whom can be the first contact for an obesity diagnosis or referral. The first two steps of the 5As framework for obesity management (ask, assess, advise, agree, and assist) aim to identify patients with obesity and start a discussion about their weight or health and obesity‐related conditions [27]. Primary care providers are well positioned to assess patients for obesity and treat them and/or refer them to specialists to address obesity and/or obesity‐related conditions [28].

In clinical practice, body mass index (BMI) is a recommended screening tool to assess obesity due to its simplicity, correlation with body fat percentage, relationship to obesity‐related conditions and mortality, and usefulness in estimating body weight and tracking population trends [29, 30, 31]. Some researchers have questioned the value of BMI as a diagnostic tool because it may be limited in predicting cardiometabolic risk factors and does not directly measure adiposity; additionally, it is not an individual measure of health and is less accurate in some populations [30, 31, 32, 33, 34]. However, a recent study of adults aged 20–59 years found that obesity, as measured by BMI, was congruent in more than 98% of individuals after a dual‐energy x‐ray absorptiometry (DEXA) scan confirmed excess adiposity [35]. Yet, another study suggested that BMI‐based cutoffs may inappropriately identify patients with normal weight who have signs of excess adiposity on imaging, such as DEXA [36].

Discussions are evolving regarding the definition and measurement of obesity, given the limitations of BMI. For example, the *Lancet Diabetes & Endocrinology* Commission recently recommended changing the definition of obesity to distinguish between preclinical and clinical obesity, the latter defined as “a chronic, systemic illness characterized by alterations in the function of tissues, organs, the entire individual, or a combination thereof, due to excess adiposity” [33]. The Commission recommends using direct measurements of body fat to confirm excess adiposity in individuals with a BMI ≤ 40 kg/m^2^, if possible, or at least one anthropometric measure in addition to BMI, such as waist circumference, waist‐to‐hip ratio, or waist‐to‐height ratio [33].

The Obesity Medicine Association (OMA) has issued a statement regarding the proposed definitions of preclinical and clinical obesity, citing concerns about redefining obesity as a risk factor rather than a disease, the potential negative impact on health insurance coverage for obesity management, and shifting away from a proactive approach to care [37]. The authors of this review echo these concerns and the recommendations of the OMA to continue to recognize obesity as a chronic disease, with a focus on preventive care *and* treatment, and the use of BMI as a screening tool, with additional measures for diagnosing obesity [37, 38].

Although the American Medical Association formally recognized obesity as a disease over a decade ago [39], many patients with obesity are not formally diagnosed [40]. A retrospective cohort study in Israel found that few individuals had a diagnosis of obesity or overweight in their medical records; those without a documented diagnosis were less likely to receive obesity care [41]. In the United States, HCPs may believe there is a lack of or lower reimbursement for obesity management, which results in underutilization of diagnostic codes [42]. An observational study of adults with obesity at 15 US health systems demonstrated that a formal obesity diagnosis in the electronic health record (EHR) significantly predicted weight loss [43].

### 3.2 Obesity Staging and Evaluation for Obesity‐Related Conditions

Once patients are diagnosed with obesity, it is important to evaluate them for the presence and severity of obesity‐related conditions (Figure 1). The Edmonton Obesity Staging System (EOSS) is a tool that can be used in the outpatient setting on its own or in combination with anthropometric measurements; it uses a scoring system to stratify risk by assessing obesity‐related risk factors, physical symptoms, functional limitations, and psychological symptoms [44]. A study in Canada determined that it was feasible to create a clinical dashboard within the EHR system to calculate the EOSS stage in adult patients in a primary care setting, providing information on a patient's stage and the severity of obesity [45]. Additional tests or evaluations may be needed, such as sleep studies for patients presenting with obstructive sleep apnea symptoms, radiological tests to diagnose knee osteoarthritis, and liver function tests to assess for metabolic dysfunction–associated steatotic liver disease or metabolic dysfunction–associated steatohepatitis. These evaluations may also expand patient access to treatment as insurance coverage for some treatments (e.g., obesity pharmacotherapies) is often available when specific comorbidities are present.

### 3.3 Treatment Initiation

The Chronic Care Model is a comprehensive approach to managing chronic diseases [46], which has been successfully implemented in pediatric obesity care, improving documentation, clinical workflows, and adherence to guidelines [47, 48]. The Comprehensive Care Model endorsed by the OMA includes four pillars of obesity treatment: nutrition therapy, physical activity, behavioral modification, and medical interventions (Figure 1) [49]. To ensure the successful management of obesity, engaging patients in shared decision‐making and tailoring treatments according to their preferences and life situations is critical—existing guides and checklists can be helpful resources [50, 51, 52]. Additionally, motivational interviewing is a patient‐centric approach to identifying individuals' motivations and commitments to behavior change that can be useful for obesity management in children and adults [53, 54].

The 5As framework for obesity management guides HCPs in helping patients with behavior change, potentially increasing obesity diagnoses and healthy behaviors, improving health, and leading to weight reduction [27, 55]. These steps include advising patients on the benefits of weight loss, discussing treatment options when patients are ready, and assisting patients by providing intensive behavioral counseling and/or referrals to specialists or obesity treatment programs [28]. The Strategies to Overcome and Prevent (STOP) Obesity Alliance proposed a 6As model, adding “arrange,” which includes scheduling follow‐up visits to assess progress toward goals, helping to coordinate care, and developing regional resources [56].

When discussing pharmacotherapy treatments with patients, consider including US Food and Drug Administration (FDA)‐approved anti‐obesity medications (AOMs) or other medications with weight‐reducing effects, such as metformin [5, 57]. Review current medications that patients take that may cause weight gain and switch to alternative non–weight‐promoting treatments, if possible [5, 57]. In the United States, navigating formulary and insurance coverage to determine a patient's eligibility and access to AOMs is an essential element of patient care—in addition to patients' ability to pay out‐of‐pocket costs—that may determine the available treatment options. Further, HCPs need to identify patients best served by a surgical approach, either as an initial option or a subsequent therapy following other treatments, including pharmacotherapy [58].

### 3.4 Treatment Continuation and Monitoring

A key component of a clinical treatment pathway for obesity is regularly monitoring weight loss, nutrition, physical activity, quality of life, improvements in obesity‐related conditions, and progress related to patient goals (Figure 1) [28, 57]. Obesity management–prioritized visits focusing on weight management have demonstrated the promotion of positive outcomes [59]. A retrospective analysis found that patients who attended all scheduled appointments postbariatric surgery had greater weight loss [60]. In addition to effective management of medication adverse effects, response to treatment should be regularly assessed, including weight loss and maintenance, improvements in anthropometric measures, blood pressure, laboratory values, and patient‐reported outcomes (e.g., quality of life, diet quality, ability to perform daily activities). For patients undergoing bariatric surgery, postsurgical weight maintenance and weight regain should also be evaluated.

Discussions regarding escalating care when treatment goals are partially or not met are key to obesity management. Regular assessments can determine whether a higher intensity of care is warranted, including referrals to specialist services, such as bariatric surgery. A study of intensive lifestyle intervention among patients with type 2 diabetes found that early response to treatment (i.e., weight change) predicted weight changes years later [61]. Moreover, a medication prescribing pathway with stopping rules and guidelines for medications can improve the success of pharmacology in obesity management, as seen in a real‐world randomized clinical trial in which significantly more individuals lost at least 15% of their body weight after 1 year compared with standard care [62].

Adjunctive aspects of obesity management should be part of a patient's treatment plan, following a team‐based care approach that includes nurses, nutritionists or dieticians, physical therapists, exercise specialists, and psychologists [63, 64]. Obesity specialists also play an important role in obesity management; American Board of Obesity Medicine (ABOM)‐certified obesity management specialists should ideally be integrated within primary care or specialty clinics. A retrospective cohort study evaluating a weight navigation program in which ABOM physicians provided treatment during weight management visits determined that this was a feasible approach that improved patient outcomes [65].

## 4 Obesity Treatment Pathways

### 4.1 Examples of Pediatric and Adult Pathway Models

Health care organizations and health systems have developed valuable insights from several obesity treatment pathways. A clinical pathway following the American Academy of Pediatrics 2023 practice guidelines outlines a process for comprehensive obesity treatment from screening to diagnosis, evaluation, and treatment [66]. Seattle Children's Hospital has a referral algorithm for managing obesity in children with a BMI above the 95th percentile [67]. A pathway from the Children's Hospital of Philadelphia provides guidance for managing obesity in the primary care and outpatient specialty care settings for patients aged 2 years or older [68]. Examples of adult clinical pathways from other countries include a detailed guide from France, which outlines specific steps such as identifying overweight and obesity, making a diagnosis, identifying a need for psychological or social support, developing a care plan, and adapting the care plan based on an individual's situation; this pathway also addresses the needs specific to older adults, women, pregnant people, and people with disabilities [69]. An algorithm from Spain offers a model with bilateral interaction between primary care and tertiary care settings [70].

Baptist Health, Northeast Florida's largest health system and employer, recently developed a comprehensive obesity treatment pathway, including core components and workflows to define medication initiation, monitoring, and continuation or discontinuation. An internal analysis revealed that approximately 70% of employees had overweight or obesity; this statistic, coupled with a sharp increase in insurance claims associated with obesity‐related conditions, highlighted the urgent need for a structured and supportive approach to weight management and overall health improvement within its workforce (P. Rama, personal communication, May 2025). The weight‐loss management protocol for employee wellness comprises an HCP referral, an employee wellness intake visit with a health coach and/or a dietitian, initiation of lifestyle therapy with patient goals and action plans, and an established cadence of follow‐up frequency and support services. The protocol includes several steps for educational sessions, discussion of treatment options, and monitoring. Additionally, there are separate steps within the care pathway for GLP‐1RA pharmacotherapy.

## 5 Considerations for Developing and Implementing Obesity Treatment Pathways

**FIGURE 2:**
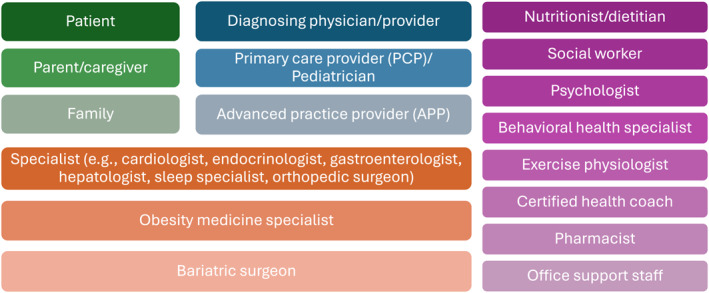
Who is involved in obesity diagnosis, treatment, and follow‐up care?

**FIGURE 3:**
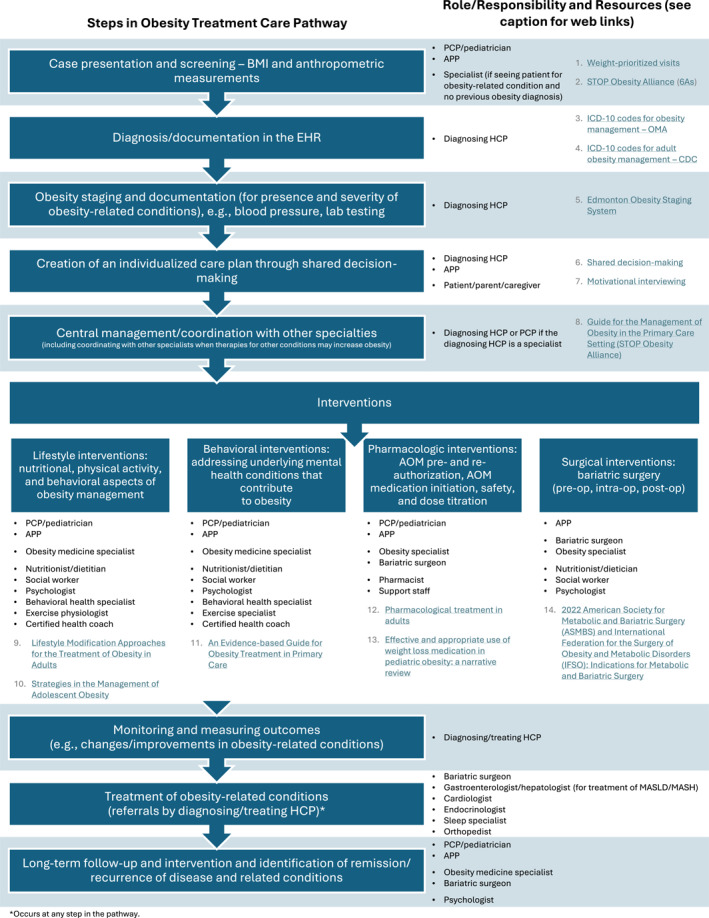
Considerations for obesity pathway development: steps, key roles, and resources. AOM, anti‐obesity medication; APP, advanced practice provider; BMI, body mass index; CDC, Centers for Disease Control and Prevention; EHR, electronic health record; HCP, health care professional; ICD‐10, International Classification of Diseases, Tenth Revision; MASLD, metabolic dysfunction‒associated steatotic liver disease; MASH, metabolic dysfunction‒associated steatohepatitis; OMA, Obesity Medicine Association; PCP, primary care provider; STOP, Strategies to Overcome and Prevent. Web links to resources: 1. https://www.aafp.org/pubs/fpm/issues/2023/1100/weight‐prioritized‐visit.html; 2. https://stop.publichealth.gwu.edu/sites/g/files/zaxdzs4356/files/2022‐02/wcw‐guide‐for‐the‐management‐of‐obesity‐in‐the‐primary‐care‐setting.pdf; 3. https://obesitymedicine.org/blog/new‐icd‐10‐codes‐for‐obesity‐treatment‐advancements‐in‐accurate‐diagnosis‐and‐care/; 4. https://www.cdc.gov/obesity/media/pdfs/2024/12/adult‐partner‐promotion‐materials‐icd‐10‐codes‐508.pdf; 5. https://www.mdcalc.com/calc/10536/edmonton‐obesity‐staging‐system‐eoss; 6. https://www.jnjmedtech.com/system/files/pdf/Obesity%20Management%20Shared%20Decision%20Making%20Tool%20012360‐210519%20.pdf; 7. https://obesitymedicine.org/blog/motivational‐interviewing/; 8. https://stop.publichealth.gwu.edu/sites/g/files/zaxdzs4356/files/2022‐02/wcw‐guide‐for‐the‐management‐of‐obesity‐in‐the‐primary‐care‐setting.pdf; 9. https://pmc.ncbi.nlm.nih.gov/articles/PMC7027681/; 10. https://pmc.ncbi.nlm.nih.gov/articles/PMC7337011/; 11. https://www.amjmed.com/article/S0002‐9343(15)00691‐9/fulltext; 12. https://pubmed.ncbi.nlm.nih.gov/25905267/; 13. https://pmc.ncbi.nlm.nih.gov/articles/PMC11294794/; 14. https://www.soard.org/article/S1550‐7289(22)00641‐4/fulltext.

The key steps of a practical obesity management care pathway follow the tenets of chronic and collaborative care models, focusing on team‐based care (Figures 2 and 3) [46, 71]. It is important to note that this information is intended as a guide to the key roles and responsibilities of HCPs within an obesity care pathway; it will necessarily vary by context, setting, and resources available in the health system. There is a lack of research on effective care pathways for modern obesity treatment. There are several steps to creating clinical treatment pathways, including evaluating current care practices, identifying guidelines, mapping the care pathway, determining the desired outcomes, engaging the team, implementing the care pathway, and evaluating the process and outcomes [72]. Successful implementation of a treatment pathway depends on several factors, including strong communication, training, monitoring, and evaluation; lack of motivation, financial reimbursement, time, and staff are among the many barriers [73, 74].

An obesity treatment pathway was developed by the Obesity Care Model Collaborative (OCMC) and implemented across 10 health care organizations or systems; it included critical components based on the structure, resources, and culture of the individual organizations, which varied considerably by type of practice or system, patient population, and staffing volume [73]. An advisory committee of obesity and quality improvement specialists guided the OCMC in developing a framework for implementing interventions for managing obesity and following treatment guidelines. The interventions spanned four domains: community, health care organization, care team, and patient or family. Gaps and challenges, recommendations, and interventions were identified and implemented across the participating organizations [73]. OCMC also developed an *Obesity Care Model Playbook* to guide organizations within primary care settings [75]. A UK public health association developed guides to aid primary care trusts in developing obesity care pathways, guiding each phase (initiation, development, implementation, and evaluation), and providing specific resources for addressing obesity in adults and children [76, 77].

**FIGURE 4:**
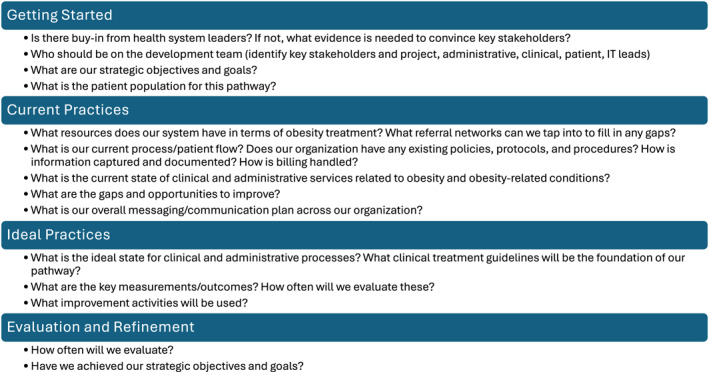
Key questions for developing obesity treatment pathways. IT, information technology.

Although developing and implementing an obesity care pathway can be challenging, a successful model can increase the quality of care and improve patient outcomes. Many questions should be considered when developing an obesity care pathway (Figure 4). For Baptist Health, the first step in developing its obesity treatment pathway was to determine the initial target population. Baptist Health employees were selected because of the direct impact on the organization's insurance costs and limited resources for nutritional and lifestyle counseling (P. Rama, personal communication, May 2025). One of the key lessons learned in developing an obesity treatment pathway for Baptist Health employees was the importance of getting buy‐in from all staff members; a physician led the initiative, which helped foster consensus among other HCPs in the health system.

Multiple challenges can hinder the implementation of obesity care pathways. Below is a list of challenges in implementing obesity treatment pathways, along with suggested strategies to address them. Research is needed to test the effectiveness of these strategies. One of the greatest challenges in managing obesity at a health system level is determining the roles and responsibilities of different providers; a qualitative study in a primary care setting identified challenges, including a lack of integration among professionals and treating obesity as a chronic condition, with the lack of standardized procedures noted to be the likely cause [78, 79]. This can result in fragmented care, unclear accountability, poor care coordination, inconsistent follow‐up, and duplicated services. Potential approaches to address these obstacles include designating a care coordinator, defining handoff and referral criteria, and using shared care plans within the EHR. Limited time and competing priorities are also challenges that may be addressed through pre‐visit questionnaires or decision aids, recorded education and teaching modules (e.g., how to perform subcutaneous injections), group counseling visits, and shared medical appointments. Provider knowledge and buy‐in regarding obesity treatment can vary. Targeted education about obesity as a chronic disease and existing and new treatment options may help to produce a cultural change to move past treating obesity using an “eat less and move more” paradigm.

Lack of or limited insurance coverage for behavioral and nutrition counseling, obesity medications, or bariatric surgery may lead to increased frustration among HCPs and patients, resulting in low adoption of the recommended treatment pathways. Ideally, a coverage assessment should occur early in the pathway, and alternative treatment branches should be created based on insurance status and health care system resources. Lack of patient engagement in care may also hinder successful implementation. Use of patient‐centered, non‐stigmatizing language, incorporation of health goals rather than weight‐centric goals, and regular follow‐up and outreach may help sustain patient engagement. It is important to identify these and other potential difficulties and proactively consider solutions that may work within the health system's parameters. Tracking metrics (e.g., referrals, retention, outcomes) and providing continuous feedback can help health systems monitor progress and address gaps. Ultimately, the success of obesity treatment pathways depends not only on evidence‐based recommendations but also on how these can be operationalized within real‐world clinical, financial, and organizational constraints.

## 6 Conclusion

The high prevalence, negative health impact, and increased health care utilization and costs of obesity make it a key focus for health care systems. Developing a care pathway for obesity can help navigate the complexity of obesity, which requires a multifaceted team approach to successfully manage the disease among patient populations of all ages successfully. Clinical practice guidelines should be leveraged to develop care pathways, providing a basis for obesity screening, diagnosis, and treatment. Care pathways must include all key elements from initial screening to continued follow‐up and assessment of patient outcomes. Obesity care pathways translate the guidance into processes tailored to a health care system or organization's culture and resources. Further research is needed to understand how obesity care pathways can be implemented within collaborative care models, such as coordinated, collocated, and integrated care [80]. Defining roles and responsibilities unique to the organization ensures that the care pathway will be helpful for clinicians and administrators; continual evaluation will keep it relevant, especially as obesity treatments evolve.

## Author Contributions

All authors contributed to drafting and revising the manuscript and approving the final version. All authors had final responsibility for the decision to submit for publication.

## Funding

Medical writing support for this paper was provided by Novo Nordisk Inc. The authors had full editorial control of the contents of the manuscript. The authors did not receive financial support related to this work.

## Conflicts of Interest

Pamela R. Rama is a speaker for Novo Nordisk. Stephanie Walsh has no conflicts of interest to disclose. Ariana Chao has served on advisory boards for Eli Lilly and Company, Novo Nordisk, and Boehringer Ingelheim, and has received grant support, on behalf of the University of Pennsylvania and Johns Hopkins University, from Eli Lilly and Company; she has also received honorarium and meeting travel support from Ro. Jason Samuels receives funding from the National Institute of Diabetes Digestive and Kidney Diseases under award number K23DK143312.

## Data Availability

The authors have nothing to report.

## References

[1] M. D. Jensen , D. H. Ryan , C. M. Apovian , et al., “2013 AHA/ACC/TOS Guideline for the Management of Overweight and Obesity in Adults,” supplement, Circulation 129, no. 25_S2 (2014): S102–S138, 10.1161/01.cir.0000437739.71477.ee.24222017

[2] C. M. Apovian , L. J. Aronne , D. H. Bessesen , et al., “Pharmacological Management of Obesity: An Endocrine Society Clinical Practice Guideline,” Journal of Cinical Endocrinology and Metabolism 100, no. 2 (2015): 342–362, 10.1210/jc.2014-3415. 25590212

[3] W. T. Garvey , J. I. Mechanick , E. M. Brett , et al., “American Association of Clinical Endocrinologists and American College of Endocrinology Comprehensive Clinical Practice Guidelines for Medical Care of Patients With Obesity,” supplement, Endocrine Practice 22, no. S3 (2016): S1–S203, 10.4158/EP161365.GL. 27219496

[4] E. Grunvald , R. Shah , R. Hernaez , et al., “AGA Clinical Practice Guideline on Pharmacological Interventions for Adults With Obesity,” Gastroenterology 163, no. 5 (2022): 1198–1225, 10.1053/j.gastro.2022.08.045. 36273831

[5] American Diabetes Association Professional Practice Committee , “8. Obesity and Weight Management for the Prevention and Treatment of Type 2 Diabetes: Standards of Care in Diabetes–2025,” supplement, Diabetes Care 48, no. S1 (2025): S167–S180, 10.2337/dc25-S008. 39651976 PMC11635032

[6] S. Wharton , D. C. W. Lau , M. Vallis , et al., “Obesity in Adults: A Clinical Practice Guideline,” Canadian Medical Association Journal 192, no. 31 (2020): E875–E891, 10.1503/cmaj.191707. 32753461 PMC7828878

[7] S. E. Hampl , S. G. Hassink , A. C. Skinner , et al., “Executive Summary: Clinical Practice Guideline for the Evaluation and Treatment of Children and Adolescents With Obesity,” Pediatrics 151, no. 2 (2023): e2022060641, 10.1542/peds.2022-060641. 36622135

[8] S. E. Hampl , S. G. Hassink , A. C. Skinner , et al., “Clinical Practice Guideline for the Evaluation and Treatment of Children and Adolescents With Obesity,” Pediatrics 151, no. 2 (2023): e2022060640, 10.1542/peds.2022-060640. 36622115

[9] T. Rotter , R. B. de Jong , S. E. Lacko , U. Ronellenfitsch , and L. Kinsman , “Clinical Pathways as a Quality Strategy,” in Improving Healthcare Quality in Europe: Characteristics, Effectiveness and Implementation of Different Strategies, eds. R. Busse , N. Klazinga , D. Panteli , and W. Quentin (European Observatory on Health Systems and Policies, 2019). 31721544

[10] R. Busse , N. Klazinga , D. Panteli , and W. Quentin , eds. Improving Healthcare Quality in Europe: Characteristics, Effectiveness and Implementation of Different Strategies [Internet] (European Observatory on Health Systems and Policies, 2019). 31721544

[11] A. C. Chiang , P. Ellis , and R. Zon , “Perspectives on the Use of Clinical Pathways in Oncology Care,” American Society of Clinical Oncology Educational Book 37, no. 37 (2017): 155–159, 10.1200/EDBK_175533. 28561702

[12] J. C. van Hoeve , R. W. M. Vernooij , M. Fiander , P. Nieboer , S. Siesling , and T. Rotter , “Effects of Oncological Care Pathways in Primary and Secondary Care on Patient, Professional and Health Systems Outcomes: A Systematic Review and Meta‐Analysis,” Systematic Reviews 9, no. 1 (2020): 246, 10.1186/s13643-020-01498-0. 33100227 PMC7586678

[13] Y. Yan , Y. Wu , A. Li , A. Yang , J. Tao , and X. Wang , “Impact of a Clinical Care Pathway Developed Through the Action Research Method on the Psychological Well‐Being and Quality of Life in Male Patients With Urethral Stricture,” Medicine 103, no. 9 (2024): e37321, 10.1097/md.0000000000037321. 38428892 PMC10906628

[14] R. Renard , “A Guide to Care Pathways With Three Examples Demonstrating Their Implications in Practice,” accessed March 28, 2025, https://awellhealth.com/blog/a‐guide‐to‐care‐pathways‐with‐three‐examples‐demonstrating‐their‐implications‐in‐practice.

[15] K. G. H. van de Pas , A. C. E. Vreugdenhil , L. Janssen , et al., “Development of a Clinical Pathway for Bariatric Surgery as an Integral Part of a Comprehensive Treatment for Adolescents With Severe Obesity in the Netherlands,” Obesity Facts 17, no. 5 (2024): 535–544, 10.1159/000539256. 38740006 PMC11458160

[16] D. A. Telem , J. Gould , C. Pesta , et al., “American Society for Metabolic and Bariatric Surgery: Care Pathway for Laparoscopic Sleeve Gastrectomy,” Surgery for Obesity and Related Diseases 13, no. 5 (2017): 742–749, 10.1016/j.soard.2017.01.027. 28416400

[17] W. H. Dietz and C. Gallagher , “A Proposed Standard of Obesity Care for all Providers and Payers,” Obesity 27, no. 7 (2019): 1059–1062, 10.1002/oby.22507. 31231954

[18] W. H. Dietz , L. S. Solomon , N. Pronk , et al., “An Integrated Framework for the Prevention and Treatment of Obesity and Its Related Chronic Diseases,” Health Affairs 34, no. 9 (2015): 1456–1463, 10.1377/hlthaff.2015.0371. 26355046

[19] J. M. Samuels , M. B. Patel , and K. D. Niswender , “Time to Rethink the Approach to Treating Obesity,” JAMA Surgery 159, no. 8 (2024): 841–842, 10.1001/jamasurg.2024.1502. 38865110

[20] R. Jackson Leach , J. Powis , L. A. Baur , et al., “Clinical Care for Obesity: A Preliminary Survey of Sixty‐Eight Countries,” Clinical Obesity 10, no. 2 (2020): e12357, 10.1111/cob.12357. 32128994

[21] National Council for Mental Wellbeing , “Toolkit for Designing and Implementing Care Pathways,” accessed March 28, 2025, https://www.thenationalcouncil.org/wp‐content/uploads/2025/02/Toolkit‐for‐Designing‐and‐Implementing‐Care‐Pathways.pdf.

[22] R. Renard , “A Comprehensive Guide to Care Pathways: Everything You Need to Know,” accessed March 28, 2025, https://awellhealth.com/blog/a‐comprehensive‐guide‐to‐care‐pathways‐everything‐you‐need‐to‐know.

[23] Department of Veterans Affairs and Department of Defense , “Clinical Practice Guideline for the Management of Adult Overweight and Obesity,” accessed March 28, 2025, https://www.healthquality.va.gov/guidelines/cd/obesity/.

[24] A. Acosta , S. Streett , M. D. Kroh , et al., “White Paper AGA: POWER ‐ Practice Guide on Obesity and Weight Management, Education, and Resources,” Clinical Gastroenterology and Hepatology 15, no. 5 (2017): 631–649.e10, 10.1016/j.cgh.2016.10.023. 28242319

[25] J. Porter and C. J. Gaskin , “Clinical Practice Guidelines for Older Adults Living With Overweight and Obesity: A Scoping Review,” Clinical Nutrition Open Science 56 (2024): 26–36, 10.1016/j.nutos.2024.04.006.

[26] A. M. Chao , A. Paul , J. V. Hodgkins , and T. A. Wadden , “A Guideline‐Directed Approach to Obesity Treatment,” Diabetes Spectrum 37, no. 4 (2024): 281–295, 10.2337/dsi24-0001. 39649692 PMC11623039

[27] E. Sturgiss and C. van Weel , “The 5 as Framework for Obesity Management: Do We Need a More Intricate Model?,” Canadian Family Physician 63, no. 7 (2017): 506–508, https://pmc.ncbi.nlm.nih.gov/articles/PMC5507219/. 28701434 PMC5507219

[28] S. L. Fitzpatrick , D. Wischenka , B. M. Appelhans , et al., “An Evidence‐Based Guide for Obesity Treatment in Primary Care,” American Journal of Medicine 129, no. 1 (2016): 115.e111–115.e117, 10.1016/j.amjmed.2015.07.015. PMC5988348 26239092

[29] G. A. Bray , K. K. Kim , and J. P. H. Wilding , “Obesity: A Chronic Relapsing Progressive Disease Process. A Position Statement of the World Obesity Federation,” Obesity Reviews 18, no. 7 (2017): 715–723, 10.1111/obr.12551. 28489290

[30] A. Romero‐Corral , V. K. Somers , J. Sierra‐Johnson , et al., “Accuracy of Body Mass Index in Diagnosing Obesity in the Adult General Population,” International Journal of Obesity 32, no. 6 (2008): 959–966, 10.1038/ijo.2008.11. 18283284 PMC2877506

[31] G. A. Bray , “Beyond BMI,” Nutrients 15, no. 10 (2023): 2254, 10.3390/nu15102254. 37242136 PMC10223432

[32] K. Sweatt , W. T. Garvey , and C. Martins , “Strengths and Limitations of BMI in the Diagnosis of Obesity: What Is the Path Forward?,” Current Obesity Reports 13, no. 3 (2024): 584–595, 10.1007/s13679-024-00580-1. 38958869 PMC11306271

[33] F. Rubino , D. E. Cummings , R. H. Eckel , et al., “Definition and Diagnostic Criteria of Clinical Obesity,” Lancet Diabetes & Endocrinology 13, no. 3 (2025): 221–262, 10.1016/s2213-8587(24)00316-4. 39824205 PMC11870235

[34] A. M. Nevill , M. J. Duncan , and T. Myers , “BMI Is Dead; Long Live Waist‐Circumference Indices: But Which Index Should We Choose to Predict Cardio‐Metabolic Risk?,” Nutrition, Metabolism, and Cardiovascular Diseases 32, no. 7 (2022): 1642–1650, 10.1016/j.numecd.2022.04.003. 35525679

[35] E. K. Aryee , S. Zhang , E. Selvin , and M. Fang , “Prevalence of Obesity With and Without Confirmation of Excess Adiposity Among US Adults,” JAMA 333, no. 19 (2025): 1726–1728, 10.1001/jama.2025.2704. 40244602 PMC12006908

[36] Z. Yao , Z. A. Dardari , A. C. Razavi , et al., “Prevalence of Clinical Obesity Versus BMI‐Defined Obesity Among US Adults: A Cohort Study,” Lancet Diabetes & Endocrinology 13, no. 8 (2025): 647–649, 10.1016/S2213-8587(25)00159-7. 41021662

[37] L. C. Alexander , “Obesity Medicine Association Raises Concerns over Lancet Commission’s New Recommendations on Obesity Diagnosis,” accessed June 11, 2025, https://obesitymedicine.org/blog/obesity‐medicine‐association‐raises‐concerns‐over‐lancet‐commissions‐new‐recommendations‐on‐obesity‐diagnosis/.

[38] N. Pennings , C. Varney , S. Hines , et al., “Obesity Management in Primary Care: A Joint Clinical Perspective and Expert Review From the Obesity Medicine Association (OMA) and the American College of Osteopathic Family Physicians (ACOFP) ‐ 2025,” Obesity Pillars 14 (2025): 100172, 10.1016/j.obpill.2025.100172. 40235850 PMC11997402

[39] L. M. Schumacher , J. Ard , and D. B. Sarwer , “Promise and Unrealized Potential: 10 Years of the American Medical Association Classifying Obesity as a Disease,” Frontiers in Public Health 11 (2023): 1205880, 10.3389/fpubh.2023.1205880. 37521999 PMC10375286

[40] L. M. Kaplan , A. Golden , K. Jinnett , et al., “Perceptions of Barriers to Effective Obesity Care: Results From the National ACTION Study,” Obesity 26, no. 1 (2018): 61–69, 10.1002/oby.22054. 29086529

[41] M. M. Kasher , S. Eizenstein , T. Cukierman‐Yaffe , and D. Oieru , “Missed Diagnosis‐A Major Barrier to Patient Access to Obesity Healthcare in the Primary Care Setting,” International Journal of Obesity 48, no. 7 (2024): 1003–1010, 10.1038/s41366-024-01514-6. 38649487 PMC11216998

[42] Avalere Health , “Barriers to Obesity Care: Diagnostic Coding, Medical Billing, and Reimbursement,” accessed July 22, 2025, https://advisory.avalerehealth.com/insights/coding‐billing‐and‐reimbursement‐barriers‐to‐obesity‐care.

[43] E. L. Ciemins , V. Joshi , J. K. Cuddeback , R. F. Kushner , D. B. Horn , and W. T. Garvey , “Diagnosing Obesity as a First Step to Weight Loss: An Observational Study,” Obesity 28, no. 12 (2020): 2305–2309, 10.1002/oby.22954. 33029901 PMC7756722

[44] A. M. Sharma , “Edmonton Obesity Staging System (EOSS),” accessed March 28, 2025, https://www.mdcalc.com/calc/10536/edmonton‐obesity‐staging‐system‐eoss.

[45] R. Swaleh , T. McGuckin , T. W. Myroniuk , et al., “Using the Edmonton Obesity Staging System in the Real World: A Feasibility Study Based on Cross‐Sectional Data,” CMAJ Open 9, no. 4 (2021): E1141–E1148, 10.9778/cmajo.20200231. PMC8673483 34876416

[46] E. H. Wagner , S. M. Bennett , B. T. Austin , S. M. Greene , J. K. Schaefer , and M. Vonkorff , “Finding Common Ground: Patient‐Centeredness and Evidence‐Based Chronic Illness Care,” supplement, Journal of Alternative & Complementary Medicine 11, no. S1 (2005): S7–S15, 10.1089/acm.2005.11.s-7. 16332190

[47] B. A. Snyder , H. He , and S. D. Duran‐Aguilar , “Improved Clinical Practice for Childhood Obesity Screening and Management,” Journal for Nurse Practitioners 20, no. 1 (2024): 104860, 10.1016/j.nurpra.2023.104860.

[48] H. Cygan , M. Reed , K. Lui , and M. Mullen , “The Chronic Care Model to Improve Management of Childhood Obesity,” Clinical Pediatrics 57, no. 6 (2018): 727–732, 10.1177/0009922817734357. 29019281

[49] A. Fitch , L. Alexander , C. F. Brown , and H. E. Bays , “Comprehensive Care for Patients With Obesity: An Obesity Medicine Association Position Statement,” Obesity Pillars 7 (2023): 100070, 10.1016/j.obpill.2023.100070.

[50] J. R. Imbus and L. M. Funk , “Shared Decision‐Making in Obesity Treatment,” in Quality in Obesity Treatment, eds. J. M. Morton , S. A. Brethauer , E. J. DeMaria , S. Kahan , and M. M. Hutter (Springer International Publishing, 2019), 155–165.

[51] Y.‐C. Lee and W.‐L. Wu , “Shared Decision Making and Choice for Bariatric Surgery,” International Journal of Environmental Research and Public Health 16, no. 24 (2019): 4966, 10.3390/ijerph16244966. 31817804 PMC6950179

[52] C. Nnonyelu , C. Onyebuchi , H. N. Okeke , et al., “Shared Decision‐Making and Obesity Management Across Healthcare Models in the United States: A Narrative Review,” medtigo Journal of Medicine 3, no. 2 (2025): e30623219, 10.63096/medtigo30623219.

[53] M. Freshwater , S. Christensen , L. Oshman , and H. E. Bays , “Behavior, Motivational Interviewing, Eating Disorders, and Obesity Management Technologies: An Obesity Medicine Association (OMA) Clinical Practice Statement (CPS) 2022,” Obesity Pillars 2 (2022): 100014, 10.1016/j.obpill.2022.100014. 37990715 PMC10661888

[54] R. Lutaud , E. Mitilian , J. Forte , et al., “Motivational Interviewing for the Management of Child and Adolescent Obesity: A Systematic Literature Review,” BJGP Open 7, no. 4 (2023): BJGPO.2022.0145, 10.3399/BJGPO.2022.0145. 37402547 PMC11176675

[55] H. M. Rowell , “Implementing the 5A’s of Obesity Management in Primary Care,” accessed March 28, 2025, https://scholarcommons.sc.edu/dnp_projects/65/.

[56] STOP Obesity Alliance , “Guide for the Management of Obesity in the Primary Care Setting,” accessed March 28, 2025, https://stop.publichealth.gwu.edu/sites/g/files/zaxdzs4356/files/2022‐02/wcw‐guide‐for‐the‐management‐of‐obesity‐in‐the‐primary‐care‐setting.pdf.

[57] S. Tucker , C. Bramante , M. Conroy , et al., “The Most Undertreated Chronic Disease: Addressing Obesity in Primary Care Settings,” Current Obesity Reports 10, no. 3 (2021): 396–408, 10.1007/s13679-021-00444-y. 34297343 PMC8300078

[58] G. E. Ames , J. R. Maynard , M. L. Collazo‐Clavell , M. M. Clark , K. B. Grothe , and E. F. Elli , “Rethinking Patient and Medical Professional Perspectives on Bariatric Surgery as a Medically Necessary Treatment,” Mayo Clinic Proceedings 95, no. 3 (2020): 527–540, 10.1016/j.mayocp.2019.09.019. 32138881

[59] E. S. Kramer , B. Deffenbacher , E. W. Staton , P. C. Smith , and J. Summers Holtrop , “The Weight‐Prioritized Visit: An Idea Whose Time Has Come,” Family Practice Management 30, no. 6 (2023): 19–25, https://pmc.ncbi.nlm.nih.gov/articles/PMC11182608/. PMC11182608 37963258

[60] J. Lujan , C. Tuero , M. F. Landecho , et al., “Impact of Routine and Long‐Term Follow‐Up on Weight Loss After Bariatric Surgery,” Obesity Surgery 30, no. 11 (2020): 4293–4299, 10.1007/s11695-020-04788-7. 32583298

[61] J. L. Unick , R. H. Neiberg , P. E. Hogan , et al., “Weight Change in the First 2 Months of a Lifestyle Intervention Predicts Weight Changes 8 Years Later,” Obesity 23, no. 7 (2015): 1353–1356, 10.1002/oby.21112. 26110890 PMC4481874

[62] D. Papamargaritis , W. Al‐Najim , J. Z. M. Lim , et al., “Effectiveness of Integrating a Pragmatic Pathway for Prescribing Liraglutide 3.0 mg in Weight Management Services (STRIVE Study): A Multicentre, Open‐Label, Parallel‐Group, Randomized Controlled Trial,” Lancet Regional Health ‐ Europe 39 (2024): 100853, 10.1016/j.lanepe.2024.100853. 38803628 PMC11129336

[63] M. Alkhatry , “Understanding and Managing Obesity: A Multidisciplinary Approach,” in Weight Loss ‐ A Multidisciplinary Perspective, ed. H. Himmerich , (2024).

[64] E. Piwowarczyk , M. MacPhee , and J. Howe , “Nurses’ Role in Obesity Management in Adults in Primary Healthcare Settings Worldwide: A Scoping Review,” Healthcare (Basel) 12, no. 17 (2024): 1700, 10.3390/healthcare12171700. 39273724 PMC11395003

[65] D. H. Griauzde , C. D. Turner , A. Othman , et al., “A Primary Care–Based Weight Navigation Program,” JAMA Network Open 7, no. 5 (2024): e2412192, 10.1001/jamanetworkopen.2024.12192. 38771575 PMC11109771

[66] CME Outfitters , “Treating Obesity as a Chronic Disease: Clinical Pathways From the 2023 American Academy of Pediatrics (AAP) Practice Guidelines,” accessed March 28, 2025, https://www.cmeoutfitters.com/wp‐content/uploads/2023/09/WC‐071‐Tools‐2.pdf.

[67] Seattle Children's , “Referral Algorithm: Obesity/BMI > 95th Percentile—All Ages,” accessed March 28, 2025, https://www.seattlechildrens.org/globalassets/documents/healthcare‐professionals/algorithms/algorithm‐obesity.pdf.

[68] Children's Hospital of Philadelphia , “Outpatient Specialty Care and Primary Care Clinical Pathway for BMI‐Based Evaluation and Management in Children,” accessed March 28, 2025, https://pathways.chop.edu/clinical‐pathway/bmi‐based‐evaluation‐management‐children‐clinical‐pathway.

[69] Haute Autorité de santé , “Care Pathway Guide: Overweight and Obesity in Adults,” accessed March 28, 2025, https://www.has‐sante.fr/upload/docs/application/pdf/2023‐03/summary_has_care_pathway_guide_overweight_and_obesity_in_adults.pdf.

[70] C. Morer , M. Úbeda , A. Ovejas , et al., “Integrative and Collaborative Approach in the Chronic Management of Obesity in Primary and Tertiary Care Setting: Vall Hebron‐SAP Muntanya Healthcare Route,” Obesity Facts 16, no. 3 (2022): 249–254, 10.1159/000528207. 36535242 PMC10826599

[71] A. Morin and S. McClendon , “Building a Collaborative Care Model: Lessons From Integrating Behavioral Health into Primary Care,” accessed June 15, 2025, https://www.manatt.com/insights/newsletters/health‐highlights/building‐a‐collaborative‐care‐model‐lessons‐from.

[72] M. Ferreira , “How to Design a Clinical Pathway?,” accessed June 26, 2025, https://www.uphillhealth.com/blog/how‐to‐design‐a‐clinical‐pathway.

[73] D. Casanova , R. F. Kushner , E. L. Ciemins , et al., “Building Successful Models in Primary Care to Improve the Management of Adult Patients With Obesity,” Population Health Management 24, no. 5 (2021): 548–559, 10.1089/pop.2020.0340. 33784483

[74] E. Seckler , V. Regauer , T. Rotter , P. Bauer , and M. Müller , “Barriers to and Facilitators of the Implementation of Multi‐Disciplinary Care Pathways in Primary Care: A Systematic Review,” BMC Family Practice 21, no. 1 (2020): 113, 10.1186/s12875-020-01179-w. 32560697 PMC7305630

[75] American Medical Group Association , “Obesity Care Model Playbook,” accessed March 28, 2025, https://www.amga.org/getmedia/f0f3f0f6‐2a91‐4984‐83f7‐7c18bbd10cc7/obesity‐care‐model‐playbook.pdf.

[76] H. Pheasant and K. Enock , “Obesity Care Pathway Support Package: Child Obesity Workbook,” accessed June 26, 2025, https://www.healthknowledge.org.uk/sites/default/files/documents/interactivel/obesity/Obesity_Child_final%2014.10.10.pdf?op=Child+Support+Pack.

[77] H. Pheasant and K. Enock , “Obesity Care Pathway Support Package: Adult Obesity Workbook,” accessed June 26, 2025, https://www.healthknowledge.org.uk/sites/default/files/documents/interactivel/obesity/Obesity_Adult_final%2014.10.10.pdf?op=Adult+Support+Pack.

[78] Ethicon US, LLC , “Obesity Management,” accessed March 28, 2025, https://www.jnjmedtech.com/system/files/pdf/Obesity%20Management%20Shared%20Decision%20Making%20Tool%20012360‐210519%20.pdf.

[79] S. Hayes , C. Wolf , S. Labbé , E. Peterson , and S. Murray , “Primary Health Care Providers' Roles and Responsibilities: A Qualitative Exploration of ‘Who Does What’ in the Treatment and Management of Persons Affected by Obesity,” Journal of Communication in Healthcare 10, no. 1 (2017): 47–54, 10.1080/17538068.2016.1270874.

[80] Healthy Minds , “Understanding Integrated Behavioral Health Care and the Collaborative Care Model,” accessed June 13, 2025, https://www.healthymindspolicy.org/research/understanding‐integrated‐behavioral‐health‐care‐and‐the‐collaborative‐care‐model.

